# CRISPR-mediated generation of a tumor-associated antigen-deficient Raji platform to investigate antigen loss in CAR-T cell therapy

**DOI:** 10.3389/fgeed.2025.1649993

**Published:** 2025-09-29

**Authors:** Aditya Ramdas Iyer, Mehwish Nafiz, Pragya Gupta, Arvinden VR, Vinodh Saravanakumar, Mohammad Sufyan Ansari, Md Shakir, Tanveer Ahmad, Sivaprakash Ramalingam

**Affiliations:** ^1^ CSIR- Institute for Genomics and Integrative Biology, New Delhi, India; ^2^ Academy of Scientific and Innovative Research (AcSIR), Ghaziabad, India; ^3^ Department of Biological Sciences and Bioengineering and Mehta Family Centre for Engineering in Medicine, Indian Institute of Technology Kanpur, Kanpur, Uttar Pradesh, India; ^4^ Multidisciplinary Centre for Advance Research and Studies, Jamia Millia Islamia, New Delhi, India

**Keywords:** CRISPR/Cas9, CAR-T cell therapy, antigen loss, antigen escape, B-cell malignancies

## Abstract

Tumor-associated antigen (TAA) loss remains a significant mechanism of resistance to chimeric antigen receptor (CAR) T cell therapy, leading to relapse in patients with B-cell malignancies and representing a major clinical challenge. Recent clinical data suggest that CD19 antigen loss triggers relapse in more than 40% of patients undergoing CD19 CAR-T cell therapy. To rigorously validate antigen loss, robust *in vitro* models that mimic the dynamic process of antigen escape are essential. However, the current absence of these models hampers our ability to fully evaluate and optimize treatment strategies. To model this clinically relevant phenomenon, we generated single (sKO), double (dKO), and triple (tKO) knockout Raji lymphoma cell lines targeting CD19, CD20, and CD22 using CRISPR/Cas9 genome editing. Initially, we established a dual-reporter cell line expressing the fluorescent marker mCherry and the bioluminescent marker Luciferase, enabling a uniform luminescence background across all the knockout cell lines before performing the CRISPR/Cas9 editing. The loss of individual or combinatorial TAAs was validated at the genomic, transcript, and protein levels. Functional co-culture assays with antigen-specific CAR-T cells showed that antigen-deficient Raji cells resisted CAR-T cell-mediated killing, closely mimicking clinical relapse. The triple knockout (tKO) model, in particular, provided a superior system compared to commonly used K562 models, as it retains the same lymphoma background while eliminating the crucial antigenic targets, thus better simulating resistance to CAR-T cell therapy. These antigen-loss models serve as valuable tools for studying mechanisms of CAR-T cell resistance and evaluating next-generation, multi-targeting CAR-T cell therapies.

## 1 Introduction

CAR-T cell therapy represents a paradigm-shifting modality in cancer immunotherapy, particularly in hematological malignancies, where unprecedented clinical responses have been achieved (Savoldo et al., 2024). It leverages genetically engineered T cells to recognize and eliminate cancerous cells expressing a specific target antigen. For B-cell malignancies, key surface antigens of B cells have been explored as therapeutic targets. Among those, CD19 has proven to be a target of choice. It is a pan B-cell marker expressed from the early stages of B-cell development and plays a pivotal role in B-cell receptor signaling, essential for B-cell survival and proliferation (Sermer et al., 2023). CD19 targeting CAR T therapy has set a new benchmark, outperforming standard-of-care regimens (Shah et al., 2019). Although high durable response rates have been achieved in patients, follow-up studies have revealed the development of cancer resistance to CAR-T cells (Cappell and Kochenderfer, 2023). Loss of surface expression of CD19 on cancer cells reduces the efficiency of CD19-specific CAR-T therapies (Ap et al., 2023). Studies on clinical trial outcomes have revealed that approximately 30%–40% of patients treated with CD19 CAR-T cells experience disease relapse (Shah et al., 2021). Among these relapsed cases, around 70% are attributed to the loss or downregulation of CD19 expression (Maude et al., 2018; Orlando et al., 2018; Saleh et al., 2023). This antigen escape mechanism poses a significant obstacle to the long-term efficacy of CD19-targeted immunotherapies.

To address this, the versatility of the CAR platform has extended well beyond CD19, with encouraging clinical activity reported for CAR-T targeting other B-cell markers like CD20 and CD22 (De Oliveira Canedo et al., 2025). CD20 (also known as MS4A1) is a mature B-cell-specific marker and is involved in calcium signaling, cell cycle regulation, and B-cell activation. It is a well-established target for monoclonal antibodies like Rituximab and CD20/CD3 bispecific engagers (Pavlasova and Mraz, 2020; Kang, 2022; Dickinson et al., 2022; Duell et al., 2024). CD22, an inhibitory coreceptor that regulates B-cell receptor (BCR) signaling, is also widely expressed in B-cell malignancies. It plays a crucial role in modulating immune responses and is targeted by therapies such as Inotuzumab ozogamicin (Clark and Giltiay, 2018). High remission rates have been achieved using CAR-T therapies singly targeting CD20 and/or CD22 or used in tandem with CD19. However, a significant proportion of patients develop cancer with lost expression of the target antigen, thereby diminishing the CAR-T therapeutic efficacy (Duell et al., 2024; Reinert et al., 2021; Shalabi et al., 2018).

To develop novel approaches for tackling antigen loss, thorough studies have become imperative for finding and validating a suitable target. To facilitate such research, suitable models that accurately reflect the phenomenon of antigen loss are needed. Several genomic and transcriptomic studies and the assessment of the antigenic profile post any antigen loss have been performed (Duell et al., 2024; Jain et al., 2022; Zhang et al., 2019). Currently, for all such studies, samples from the disease-relapsed patients are being used. While these may be a suitable system, the availability of such samples is limited. It places undue dependence on the CAR-T trials and significantly extends the waiting time for both trial initiation and subsequent follow-up studies, thereby hampering research progress. Furthermore, patient cells, being primary, are difficult to maintain in culture for a long time, making them less suitable models for validating any novel antibody or CAR against antigen loss.

Numerous studies on B-cell malignancies have been done using Raji, which is a cell line derived from human Burkitt lymphoma (Fousek et al., 2021; Hu et al., 2020; Schneider et al., 2021). Its consistent expression of key B-cell markers including CD19, CD20, and CD22, makes it particularly suited for investigating antigen-targeted immunotherapies. Their consistent growth kinetics and ease of culture render them ideal for screening and functional assays. While this cell line may be an ideal model, however, it does not replicate the disease phenotype of antigen loss.

To our knowledge, there are currently no well-characterized cellular models that accurately represent antigen loss, limiting the ability to comprehensively evaluate and optimize treatment strategies. To confront this limitation, we used the CRISPR/Cas9 mediated genome editing approach to generate antigen knockouts (KO) in the Raji cell line. These KO lines were designed to model antigen loss by selectively disrupting the expression of one or more target antigens. These KO lines were generated in a clonal population of Raji cells engineered to stably co-express firefly luciferase and the fluorescent protein mCherry. The presence of luciferase enabled quantitative and high-throughput assessment of CAR-T mediated cytotoxicity using luminescence-based assays, while mCherry served as a fluorescent marker for tracking the transgene expression and also aided in fluorescence-based cell sorting. This dual-reporter system of luciferase-expressing Raji cell line (Raji Luc-WT) provided a uniform luminescent background, ensuring consistency across all KO variants, and facilitating robust comparison of CAR-T responses under varying antigen expression conditions. By systematically removing one or more antigens, these KO Raji cell lines present a suitable model to mimic the antigen loss. The present study aims to provide a comprehensive characterization of the generated KO Raji cells, confirming their antigen loss at the gene, transcript, and protein levels and validating their utility in CAR-T cytotoxicity assays. These KO models can pave the way to assess how CAR-T cells respond to varying antigen expression patterns and aid in evaluating the potential benefits of multi-targeting strategies. Our model systems will contribute to the optimization of CAR-T therapies designed to counteract antigen escape, ultimately improving treatment outcomes for patients with B-cell malignancies.

## 2 Materials and methods

### 2.1 sgRNA design and validation

Single guide RNAs (sgRNAs) targeting CD19, CD20, and CD22 were designed using the online tool CHOPCHOP (Labun et al., 2019). The sgRNAs with high specificity and minimal off-targets were selected. Off-target sites were predicted using COSMID, and only sgRNAs with minimal predicted off-target activity were selected for cloning. The chosen sgRNAs were cloned into the pSpCas9(BB)-2A-Puro (pX459) vector (Addgene-62988). To validate sgRNA activity at endogenous target sites, HEK293T cells were transfected with the CRISPR/Cas9 constructs, and genomic DNA was extracted 72 h post-transfection. Target regions were PCR-amplified and analyzed using the T7 endonuclease I (T7E1) assay to detect CRISPR-induced indels. The sgRNA locations, sequences, and genotyping primers used in this study are provided in Supplementary Figure S2A and Supplementary Table S1.

### 2.2 Raji culture

Raji cells were cultured in RPMI-1640 medium (ThermoFisher Scientific) supplemented with 10% fetal bovine serum (FBS) (ThermoFisher Scientific) under standard conditions (37 °C, 5% CO_2_). Cells were maintained at a density between 0.2 × 10^6^ and 1 × 10^6^ cells/mL and passaged every 2–3 days by diluting with fresh medium. Cell viability was assessed using Trypan blue exclusion and counted using a hemocytometer. For cryopreservation, cells were resuspended in freezing medium (90% FBS + 10% DMSO) at 1–5 × 10^6^ cells/mL, aliquoted into cryovials, and stored at −80 °C overnight before transfer to liquid nitrogen. Thawing was performed by rapid warming in a 37 °C water bath, followed by centrifugation and resuspension in fresh culture medium.

### 2.3 Luciferase line generation

Lentiviral particles were produced by co-transfecting HEK293T cells with pCDH-EF1a-eFFly-mCherry (Addgene-104833), the packaging plasmid pCMV-dR8.2 dvpr (Addgene-8455), and the envelope plasmid pMD2.G (Addgene-12259) using PEI Prime™ linear polyethylenimine (Sigma-Aldrich) as the transfection reagent. HEK293T cells were incubated at 37 °C, 5% CO_2_, and viral supernatants were collected at 48 and 72 h post-transfection. The supernatants were filtered through a 0.45 µm filter, concentrated using Lenti-X Concentrator (Takara), and stored at −80 °C until use. Raji cells were transduced with lentiviral particles and maintained in culture for 48 h before bulk sorting based on mCherry fluorescence using BD FACSAria™ III flow cytometer. Single-cell sorting was then performed on the enriched bulk-sorted population to obtain clonal populations. Stable luciferase expression in the clonal lines was confirmed by luciferase assay using D-luciferin (Revvity) and qRT-PCR for WPRE, Luciferase, and mCherry genes.

### 2.4 CRISPR/Cas9-mediated genome editing

Genome editing was performed in Raji Luc WT cells to generate CD19, CD20, and CD22 knockouts using CRISPR/Cas9. sgRNAs previously screened and validated in HEK293T cells were used to create deletions in both alleles. Cells were electroporated using the Neon™ Electroporation System (ThermoFisher Scientific) with the following settings: 1,600 V, 10 ms, 3 pulses in Buffer R. Post-electroporation, cells were maintained in RPMI-1640 medium supplemented with 10% FBS, 1× MEM Non-Essential Amino Acids (MEM-NEAA), and 1× Sodium Pyruvate to support cell recovery and viability. Edited cells were bulk-sorted based on GFP fluorescence using flow cytometry using BD FACSAria™ III flow cytometer. The genomic deletion was confirmed by PCR, detecting the expected gap band. Single-cell sorting was performed to isolate clonal populations, screening was done by detecting the gap band and biallelic deletions were validated by Amplicon sequencing.

### 2.5 Cell viability assay

Cell viability was assessed using the AlamarBlue Cell Viability Reagent (Invitrogen). Raji cells were seeded in 96-well plates at a density of 10,000 cells per well in 90 µL of complete RPMI medium and AlamarBlue reagent (10% v/v) was added to each well and cells were incubated for 2 h at 37 °C with 5% CO_2_. Fluorescence was measured at 570 nm excitation and 585 nm emission using a microplate reader (Tecan Life Science). Background values from medium-only controls were subtracted, and relative viability was calculated against Raji WT.

### 2.6 Cell proliferation assay

Cells were seeded in 24-well plates at a density of 1 × 10^5^ cells per well in complete RPMI medium and incubated at 37 °C with 5% CO_2_. Cells were harvested and live cells were counted with trypan blue staining at 24, 48, 72, and 96 h using a hemocytometer. Proliferation was normalized to Raji WT counts at each time point.

### 2.7 qRT-PCR

Total RNA was extracted using TRIzol^TM^ (Thermo Fisher Scientific). After DNase treatment (Thermo Scientific), 1ug of extracted RNA was reverse transcribed into cDNA using High-Capacity cDNA Reverse Transcription Kit (Applied Biosystems) as per manufacturer’s protocol. Real-time qPCR was performed using TB Green Premix Ex Taq II (Takara), with GAPDH as housekeeping control. Fold change was determined using the ΔΔCt method. The primer details are provided in Supplementary Table S1.

### 2.8 Flow cytometry

For surface staining of Raji, cells were washed twice with PBS containing 0.5% BSA and resuspended at 1 × 10^6^ cells per 100 µL in staining buffer. For CD19 and CD22 staining, cells were incubated with CD19-PE (Miltenyi Biotec) and CD22-APC (Miltenyi Biotec) antibodies for 30 min at 4 °C in the dark. For CD20 staining, cells were first incubated with Rabbit anti-CD20 (Abcam) for 30 min at 4 °C, washed, and then stained with a FITC-conjugated secondary antibody (Abcam) for 30 min at 4 °C in the dark.

For T cell flow cytometry, all the antibodies were from Biolegend, unless otherwise stated. 0.5 × 106 cells were harvested and washed in 0.5% BSA. Staining was CD3-PE, CD4-FITC, CD8-FITC, CD14-APC, CD56-APC, CD19-APC, CD20-APC, CD22-APC (Miltenyi Biotec). For activation marker analysis, T cells were harvested 48 h post-activation and stained with CD3-PE, CD25-FITC, and CD69-FITC. For assessment of CAR expression, 0.5 × 106 cells were harvested and washed with 0.5% BSA. CAR19-BB and CAR22-BB were stained with Biotinylated CAR19 and CAR22 detection reagents (Miltenyi Biotec) respectively, followed by APC-conjugated streptavidin. Untransduced cells were used as a negative control. All the staining procedures were performed according to the manufacturer’s protocol. Data was acquired using a BD Accuri™ flow cytometer and analyzed using FlowJo v10.10 software. The complete list of antibodies used in the study is given in Supplementary Table S3.

### 2.9 Immunofluorescence

Cells were serum-starved for 30 min to facilitate attachment to coverslips, then washed with PBS and fixed using 4% paraformaldehyde (PFA) for 10 min at room temperature (RT). After fixation, cells were washed twice with PBS, and blocking was performed using 5% BSA in PBS for 1 h at RT to prevent nonspecific binding. For CD19 and CD22 staining, cells were incubated with CD19-PE (Miltenyi Biotec) and CD22-APC (Miltenyi Biotec) overnight at 4 °C in the dark. For CD20 staining, cells were incubated with Rabbit anti-CD20 (Abcam) overnight at 4 °C, washed with PBS, and then incubated with a FITC-conjugated secondary antibody for 1 h at RT in the dark. After staining, coverslips were washed with PBS, mounted using antifade mounting medium with DAPI (ThermoFisher Scientific), imaged using a confocal microscope (Leica Stellaris), and analyzed using Leica LAS X software.

### 2.10 CAR construction

The scFvs used for CAR construction were derived from FMC63 (CD19), Leu16 (CD20), and m971 (CD22). These were fused to a CD8α hinge/transmembrane domain, followed by 4-1BB and CD3ζ intracellular signaling domains. Coding sequences were cloned downstream of the EF1ɑ promoter and upstream of the Woodchuck Hepatitis virus Posttranscriptional Regulatory Element (WPRE). GFP was added to the construct as a reporter marker, separated by a P2A peptide. The transgene was then cloned into an empty lentiviral backbone vector.

### 2.11 Primary T cell culture and CAR transduction

The study was approved by the Institutional Human Ethics Committees at the Institute of Genomics and Integrative Biology (CSIR-IGIB/IHEC/2023-24/06). Blood samples were collected from three different healthy donors and peripheral blood mononuclear cells (PBMCs) were isolated using Ficoll-Paque density gradient centrifugation. CD4^+^ and CD8^+^ T cells were purified from PBMCs using magnetic-activated cell sorting (MACS) with CD4 and CD8 MicroBeads (Miltenyi Biotec). Purified T cells were cultured in TexMACS Medium (Miltenyi Biotec) supplemented with 500 U/mL IL-7 (Miltenyi Biotec) and 40 U/mL IL-15 (Miltenyi Biotec) and maintained at 37 °C in a humidified incubator with 5% CO_2_. For activation, T cells were stimulated using Human TransAct (Miltenyi Biotec) according to the manufacturer’s instructions. Twenty-four hours after activation, T cells (0.5 × 106 cells per well) were transduced with lentiviral CAR particles at a multiplicity of infection (MOI) of 5 by directly adding the virus to the culture. Media was replenished every 2–3 days, and transduced T cells were expanded for further experiments. The list of cytokines used is given in Supplementary Table S3.

### 2.12 *In vitro* co-culture

Prior to co-culture, cytokines were removed by washing CAR-T cells with fresh medium. Raji cells (10,000 cells per well) were seeded in 96-well plates, and CAR-T cells were added at effector-to-target (E:T) ratios of 1:1, 2.5:1, 5:1, and 10:1. Co-culture was performed in a 1:1 ratio of TexMACS Medium and RPMI-1640 + 10% FBS. Cells were incubated overnight at 37 °C in a humidified incubator with 5% CO_2_. Cytotoxicity was assessed using a luciferase-based killing assay and luminescence was measured using a Microplate Spectrophotometer (Tecan Life Science).

### 2.13 Off-target editing efficiency analysis

The off-target regions of sgRNAs used to generate CD19, CD20, and CD22 knockouts were identified using COSMID tool (https://crispr.bme.gatech.edu/). Editing efficiencies of the top 5 off-target regions for each sgRNAs were assessed using NGS-based amplicon sequencing. Briefly, the amplified PCR products were tagmented and sample barcodes were added before sequencing on MiSeq or Novaseq6000 platforms. The raw reads obtained were trimmed using Trimmomatic and then aligned using BWA-MEM against the hg38 reference genome. Editing efficiencies were calculated using in-house R scripts looking at % InDels near the sgRNA cut site. The complete dataset has been deposited in the BioProject database and is available at http://www.ncbi.nlm.nih.gov/bioproject under BioProject ID - PRJNA1251797.

### 2.14 Statistical analysis

Statistical analysis of data was performed with GraphPad Prism Software (Version 9.5.0, GraphPad Software Inc., United States) using appropriate statistical tests as indicated in individual figure legends. Experiments were performed in triplicates and standard deviations were represented as error bars in all figures. P-values <0.05 were considered statistically significant.

## 3 Results

### 3.1 Raji Luc WT cells show stable integration of the Luciferase-mCherry gene while maintaining parental cell characteristics

**FIGURE 1:**
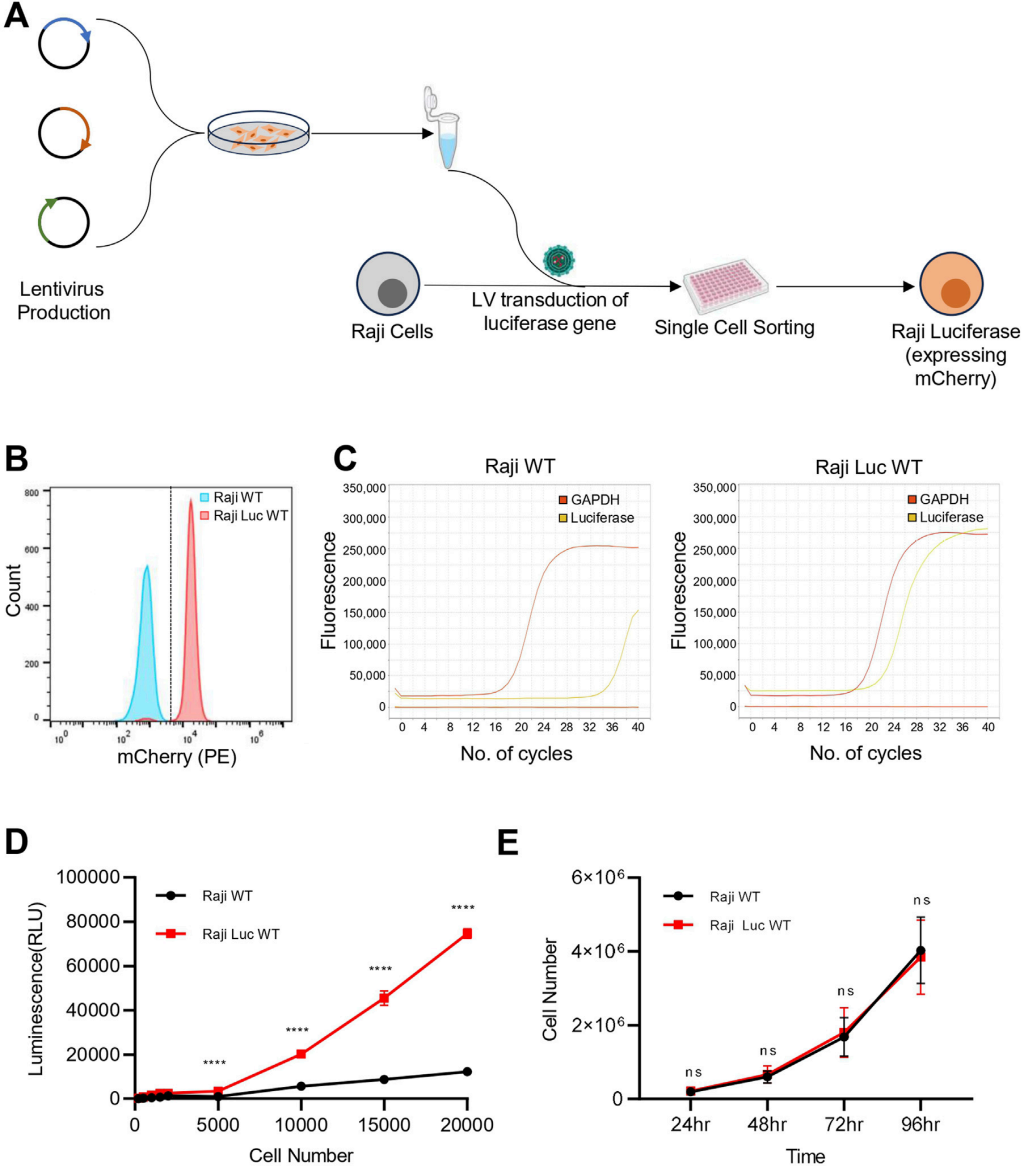
Raji-Luc-WT stably express functional luciferase-mCherry and retain parental features. **(A)** Schematic representation of the experimental workflow for generating Raji-Luciferase line from Raji WT. **(B)** Flow cytometry analysis confirming stable expression of mCherry in the Raji-Luc-WT. **(C)** qRT-PCR analysis showing robust luciferase gene expression in Raji-Luc-WT cells while no detectable amplification was observed in untransduced Raji-WT cells (Ct > 35). GAPDH was used as a housekeeping control. Data shown is the representative of three independent experiments. **(D)** Luciferase activity assay demonstrating dose-dependent luminescence in Raji-Luc-WT cells across increasing cell numbers. Parental Raji-WT cells showed negligible luminescence under the same conditions, confirming functional transgene expression. **(E)** Proliferation assay comparing Raji-Luc-WT and parental Raji-WT cells over 96 h. Both cell lines exhibited similar growth kinetics. Data plotted represents mean ± SD. Statistical analysis was performed using multiple unpaired t-tests with Holm–Šidák correction for multiple comparisons. ****p < 0.0001; ns = not significant.

The experimental strategy for generating the Raji-mCherry-Luciferase line (hereafter; Raji-Luc-WT) is outlined in Figure 1A. Lentiviral vector encoding mCherry and Luciferase was made and cells were transduced at a defined MOI. Following transduction, a clonal population was generated, and mCherry expression was confirmed in the population using fluorescence imaging and flow cytometry (Figure 1B; Supplementary Figure S1A). To check for the presence of the transgene in the transduced cells, we evaluated the relative mRNA expression of the Luciferase gene by qRT-PCR (Figure 1C; Supplementary Figure S1B). In Raji-Luc-WT cells, a Ct value of ∼ 22 was obtained for the luciferase gene, indicating stable incorporation and expression of the transgene. In contrast, the untransduced parental Raji cells (Raji-WT) showed no detectable amplification, with Ct values >35, indicating the absence of luciferase transcript levels. To ascertain whether the luciferase gene is yielding a functional protein, we performed an *in vitro* dose-dependent luciferase assay (Figure 1D). The assay was done with varying cell densities of Raji-Luc-WT and Raji-WT cells. Upon addition of the luciferin substrate, Raji-Luc-WT cells exhibited significant luminescence at all the tested cell numbers, with signal intensity increasing proportionally with the cell count. In contrast, Raji-WT cells displayed negligible luminescence across all conditions, confirming the absence of luciferase activity. These results validate the functional expression of the luciferase transgene in the Raji-Luc-WT cell line and its utility for quantitative luminescence-based assays.

Given that the lentiviral integration events in the genome are random, we checked if the luciferase gene integration is affecting cell division. Proliferation analysis revealed that Raji-Luc-WT exhibited a similar growth pattern as that of parental Raji WT when monitored for over 96 h (Figure 1E). This indicates that luciferase gene integration did not result in any significant changes to cell proliferation.

### 3.2 Raji KO lines retain proliferation and viability following CRISPR-mediated genomic deletions

To develop a robust model system for antigen escape, we generated Raji-Luc cell lines with single, double, and triple knockouts of CD19, CD20, and CD22 using CRISPR/Cas9-mediated genome editing. Multiple sgRNAs targeting the exonic regions of CD19, CD20, and CD22 genes were designed and validated for their cleavage efficiency in HEK293T cells using T7 endonuclease I assay (Supplementary Figures S2A–D). To generate a knockout, dual sgRNAs for each gene were used simultaneously to create a deletion in the CD19, CD20, and CD22 gene locus.

**FIGURE 2:**
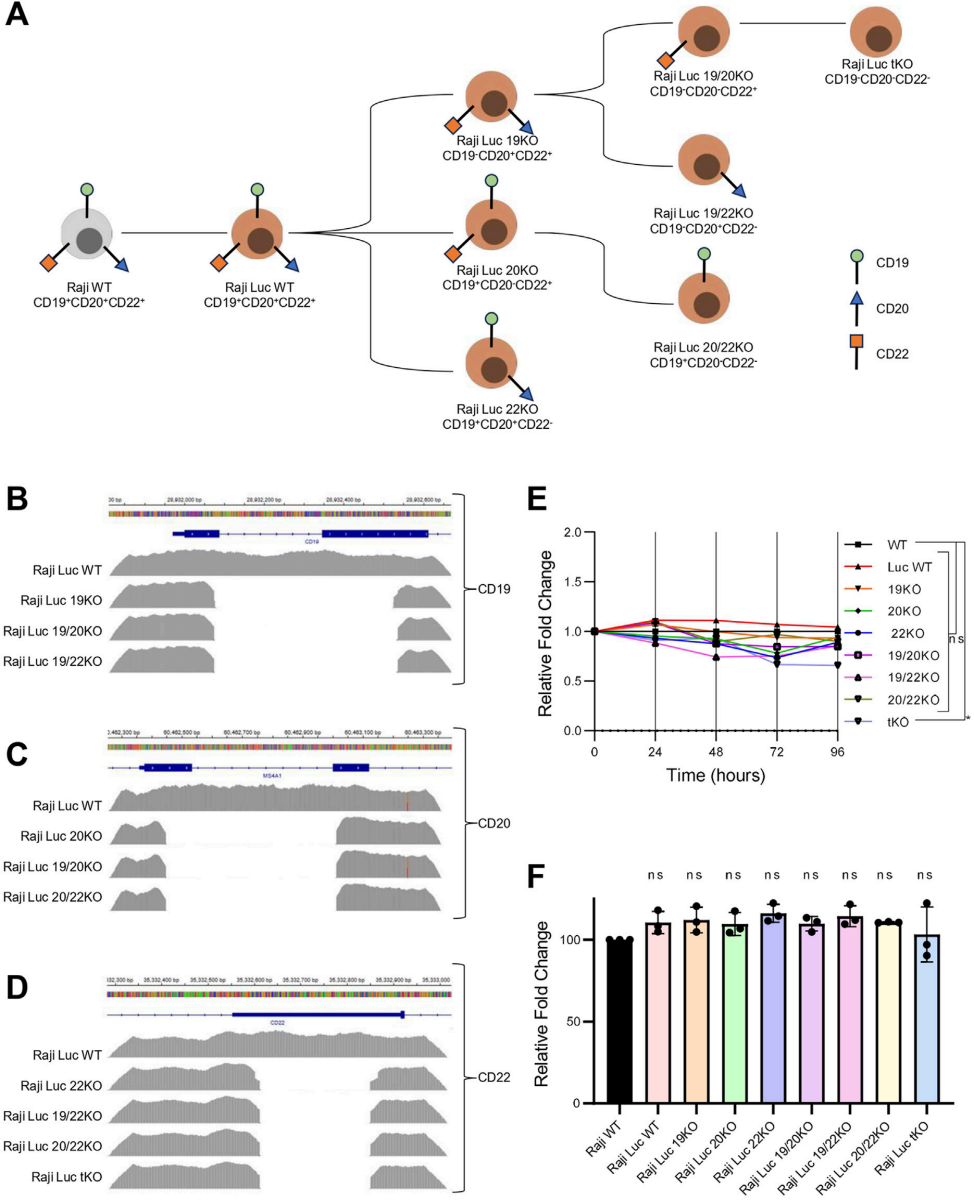
Genomic deletion of target antigens in Raji-Luc lines does not alter proliferation or viability. **(A)** Schematic representation of CRISPR-Cas9–mediated strategy to generate single, double, and triple knockout (KO) Raji-Luc cell lines. **(B–D)** Amplicon sequencing data showing genomic deletions at the CD19 **(B)**, CD20 **(C)**, and CD22 **(D)** loci in the respective KO lines, confirming targeted gene disruption. **(E)** Proliferation assay comparing all the Raji-Luc KO lines and control Raji-Luc-WT over 96 h. Data plotted as fold change normalized to Raji WT. **(F)** Viability analysis after 2 h incubation with AlamarBlue reveals no significant differences between Raji WT, Raji-Luc WT, and KO lines. Statistical analysis was performed using ordinary one-way ANOVA followed by the Dunnett’s multiple comparisons test. *, p < 0.05; ns = not significant.

Initially, Raji-Luc-WT was used to generate single knockout lines by introducing sgRNAs targeting CD19, CD20 or CD22. The clonal population of the CD19 KO line was then used to generate CD19/CD20 and CD19/CD22 double knockouts, while the CD20 KO line was used for the generation of CD20/CD22 double knockout. The CD19/CD20 double knockout was subsequently used to generate the triple knockout line lacking all three antigens (Figure 2A). Genomic sequence alterations in the knockout lines were evaluated by amplicon sequencing. The alignment of the amplicon sequence to the reference sequence confirmed the deletion of the genomic region in the knockout lines (Figures 2B–D). Off-target effects of sgRNAs used in the study were computationally predicted using the COSMID (Cradick et al., 2014), and the top five predicted off-target sites were assessed by NGS-based amplicon sequencing (Supplementary Table S2). No significant off-target mutations were detected for any of the sgRNAs, except for sgRNA3 targeting CD19, which showed a minimal off-target effect (0.18%) in one predicted region. This off-target site was located in an intergenic region with no known regulatory elements, suggesting a negligible functional impact (Supplementary Figure S3).

Next, we evaluated the proliferation potential and viability of the Raji KO lines. Proliferation was monitored over a 96-h period, and no significant differences were observed among any of KO cell lines compared to the parental Raji WT (Figure 2E). Cell viability was assessed using the AlamarBlue assay, which showed that Raji Luc WT and all the Raji KO lines have comparable viability to that of parental Raji WT cells (Figure 2F). These results suggest that neither the integration of the luciferase transgene nor the introduced genetic alterations in KO lines affected the cell proliferation or viability.

### 3.3 Raji KO lines lack expression of both the target transcript and protein, exhibiting a phenotype consistent with the antigen loss

**FIGURE 3:**
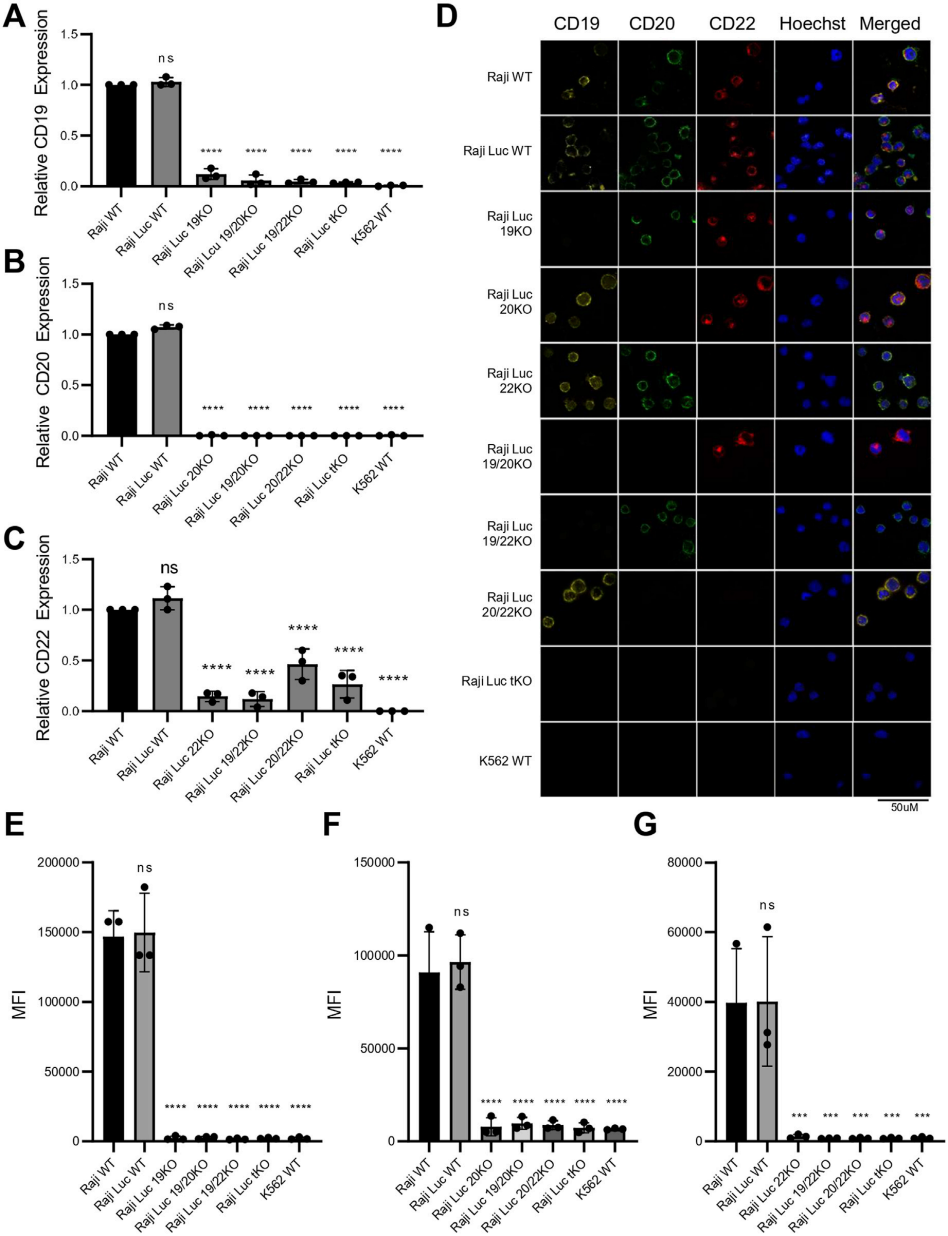
Raji knockout lines generated via CRISPR-Cas9 exhibit complete loss of CD19, CD20, and CD22 transcript and protein expression. **(A–C)** Transcript level analysis via qRT-PCR revealed significant reduction in the mRNA levels of **(A)** CD19, **(B)** CD20 and **(C)** CD22. K562 was used as CD-marker negative control. Gene expression was normalized to Raji WT and GAPDH was used as an internal control. Data plotted as fold change (mean ± SD, n = 3). **(D)** Immunofluorescence staining for CD19 (yellow), CD20 (green), CD22 (red), and Hoechst (blue) in all Raji cell lines and K562. Loss of surface antigen staining is evident in all corresponding knockout lines. Images are representative of three independent experiments. Scale bar = 50 μm. **(E–G)** Flow cytometry analysis of surface CD19 **(E)**, CD20 **(F)**, and CD22 **(G)** expression in nonpermeabilized Raji-derived knockout cells and K562 control. Data represent mean ± SD of fluorescence intensity (MFI) from three independent experiments, confirming complete loss of surface expression of the respective target antigen in each knockout line. Statistical analysis was performed using ordinary one-way ANOVA followed by the Dunnett’s multiple comparisons test. ****, p < 0.0001; ***, p < 0.001; ns = not significant.

To ensure the CRISPR/Cas9-mediated genetic alterations resulted in the effective elimination of the target antigens, we evaluated both transcript and protein levels of CD19, CD20, and CD22. Transcript analysis by qRT-PCR revealed that the expression levels of all three antigens in Raji-Luc WT were comparable to those in the parental Raji WT (Figures 3A–C), indicating that luciferase gene integration did not interfere with the endogenous gene expression of CD19, CD20 or CD22. The transcript levels of CD19 and CD20 were nearly undetectable in the Raji 19KO and Raji 20KO respectively confirming successful gene disruption at the mRNA level resembling the negative control, K562. For CD22, although the transcript levels were not absent in Raji 22KO, they were significantly reduced as compared to the parental controls.

Given that all three target antigens are expressed on the cell surface, we detected their expression using Immunofluorescence and Flow Cytometry on non-permeabilized cells. Immunofluorescence staining of the Raji cell lines using antibodies specific to CD19, CD20, and CD22 confirmed the absence of the corresponding antigen in each KO line. Notably, the expression of non-target antigens remained unaffected, demonstrating the specificity of the knockouts and confirming that other surface markers were not altered (Figure 3D). Flow cytometric analysis revealed clear peak shifts for CD19, CD20, and CD22 in both Raji WT and Raji-Luc WT, confirming normal surface expression of all three antigens. In contrast, CD19 KO, CD20 KO, and CD22 KO cell lines showed no detectable peak shift and a corresponding reduction in MFI to baseline levels upon staining with the respective antigen-specific antibodies, indicating a complete loss of surface expression of the target antigens. The fluorescence profiles of the knockout lines were indistinguishable from the negative control, K562, further validating the successful elimination of CD19, CD20, and CD22 in their respective KO cell lines (Figures 3E–G; Supplementary Figure S4). Elimination of the expression of target antigens from the cell surface results in a phenotypic resemblance to cancer cells exhibiting antigen loss.

### 3.4 Raji KO lines serve as robust models of antigen loss, exhibiting resistance to antigen-specific CAR-T cell cytotoxicity

**FIGURE 4:**
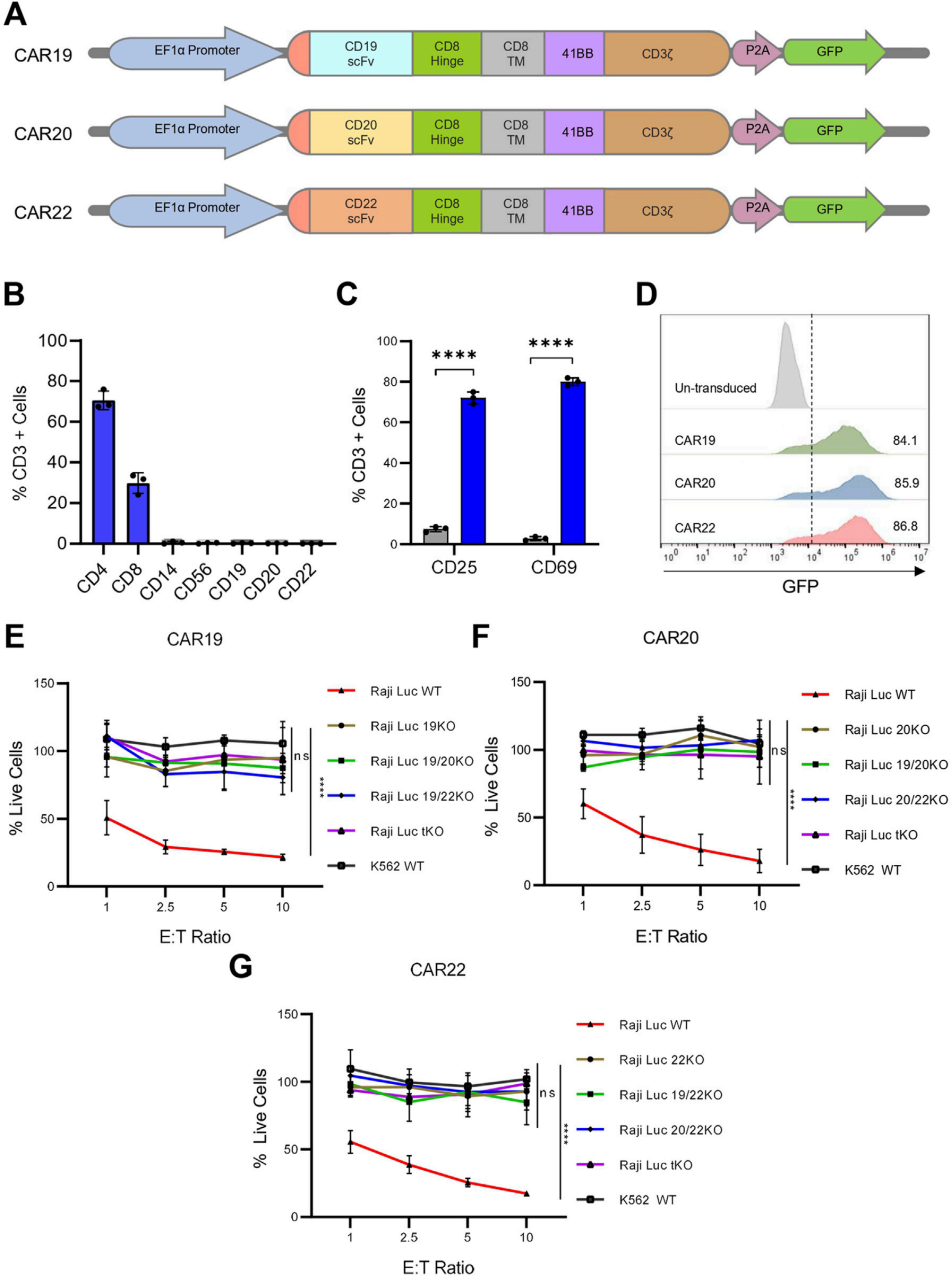
Raji KO lines model antigen loss and resist CAR-T cell–mediated cytotoxicity. **(A)** Schematic representation of CAR constructs targeting CD19, CD20, and CD22. **(B)** Flow cytometric analysis of isolated T cells confirmed the expression of T cell–specific markers and negligible expression of non–T cell markers. **(C)** Expression of T-cell activation markers on isolated T cells post 48 h of activation with CD3/CD28 beads. **(D)** Flow cytometry of GFP expression in T cells 48 h post-transduction. Histogram shown from one of three independent transductions using T cells from three different donors. **(E–G)** Viability of Raji KO lines assessed by bioluminescence following 16 h co-culture with CAR T cells expressing CAR19 **(E)**, CAR20 **(F)** or CAR22 **(G)** at indicated effector-to-target (E:T) ratios. Percent live cells was calculated based on luminescence signal relative to target-only control and normalized to untransduced controls. CAR-T cells selectively eliminated WT Raji cells expressing the corresponding target antigen, while respective single, double, and triple KO lines remained resistant. K562 cells served as antigen negative control. Data are presented as mean ± SD for three independent biological replicates. Statistical analysis was performed using multiple unpaired t-tests (one per row) with Holm–Šidák correction for multiple comparisons **(C)** or by comparing area under the curve for each cell line group in an ordinary one-way ANOVA with Dunnett’s multiple comparisons test **(E–G)**. ***, p < 0.001; ****, p < 0.0001; ns = not significant.

To assess the functional relevance of our generated Raji KO cell lines, we performed CAR-T cell-based cytotoxicity assays. The scFvs targeting CD19, CD20, and CD22 were assembled into a lentiviral vector, with CD8α hinge and transmembrane domain, a 4-1BB costimulatory domain, and a CD3ζ activation domain (hereafter referred to as CAR19, CAR20, and CAR22 respectively) (Figure 4A; Supplementary Figures S5A–C). Human primary T cells were isolated from three different healthy donor peripheral blood mononuclear cells (PBMCs) using density gradient centrifugation. Flow cytometric analysis of isolated T cells was performed and activation was done with CD3/CD28 beads revealed a composition of ∼70% CD4^+^ and ∼30% CD8^+^ populations. Additionally, almost negligible expression of non-T cell markers like CD19, CD20, CD22, CD56, and CD14 was detected, confirming a highly purified T cell population (Figure 4B). Following 48 h of activation, primary T cells showed significant upregulation of activation markers like CD25 and CD69, with ∼72% and ∼80% of cells expressing these activation markers, respectively. In contrast, the unactivated T cells showed minimal expression of CD25 and CD69 (∼7% and ∼3% respectively) (Figure 4C).

The activated T cells were transduced with lentivirus encoding CAR19, CAR20 or CAR22 constructs expressing a GFP reporter molecule. Transduction was initially confirmed by detecting GFP fluorescence in T cells (Supplementary Figures S6A,B). Flow cytometric analysis revealed high transduction efficiencies for all three CAR constructs, with each construct showing >80% GFP-positive T cells (Figure 4D; Supplementary Figure S6C). CAR surface expression and specificity were confirmed using scFv-specific antibodies (Supplementary Figures S6D,E). The CD19 scFv antibody bound exclusively to CAR19-T cells, while the CD22 scFv antibody recognized only CAR22-T cells. No cross-reactivity was observed, validating both the surface CAR expression and target specificity.

To evaluate the cytotoxic effect of these CAR constructs on the Raji KO model lines, we used different effector-target (E:T) cell ratios for the co-culture of each of the CAR constructs with the knock-out lines (Figures 4E–G). At 1:1 E:T ratio, the percentage of live Raji Luc WT cells were ∼50%, ∼60%, and ∼55% when co-cultured with CAR19, CAR20, and CAR22, respectively for 16 h. Increasing the E:T ratio to 10:1 led to a marked reduction in the percentage of live cells, with only ∼20%, ∼17%, and ∼18% of target cells remaining, respectively, indicating robust and dose-dependent cytotoxicity by all three CAR constructs. Untransduced (UT) T cells were included as a control, and minimal to no target cell killing was observed when cocultured with WT, KO lines or K562 cells. Negligible cytotoxicity was observed when any of the three CAR-T cells were co-cultured with K562 cells, maintaining ∼100% live cells even at the highest E:T ratio of 10:1, confirming the antigen-specific nature of CAR-T-mediated cytotoxicity. Notably, minimal CAR-T cell-mediated cytotoxicity was observed when co-cultured with the KO lines lacking the specific target antigen. CAR19 T cells, when co-cultured with Raji 19KO cells, the percentage of live cells decreased to a minimum of ∼95% at E:T ratio of 10:1, confirming that CAR19 failed to eliminate CD19-null cells (Figure 4E). A similar trend was observed for CAR20 and CAR22, where CD20 null cell lines were resistant to killing by CAR20 and CAR22, respectively (Figures 4F,G). The percentage of live cells dropped to a minimum of ∼95% and ∼85% for CAR20 and CAR22, respectively, at a maximum E:T ratio of 10:1. In conclusion, the Raji KO cell lines perfectly model the antigen loss scenario and show resistance to CAR-T cell-mediated cytotoxicity.

## 4 Discussion

CD19-directed CAR-T cells have shown promising results in patients with refractory and relapsed B cell malignancies, where other conventional treatment methods like chemotherapy, and radiation therapy have failed (Dagar et al., 2023; Davila and Brentjens, 2016; Westin et al., 2023; Abramson et al., 2020). Despite the impressive efficacy of CD19 CAR-T cell therapy, a significant number of patients (up to 50%) experience disease progression due to cancer resistance mechanisms. One emerging molecular mechanism of CD19 CAR T cell relapse is CD19 antigen escape with expression below the threshold required for effective CAR T cell activation such as antigen loss or reduced CD19 expression below the threshold required for effective CAR-T-cell activation (Shah and Fry, 2019). The emergence of antigen loss as a mechanism of resistance poses significant challenges to the long-term efficacy of CD19 CAR-T cell therapies (Shah and Fry, 2019; Lin et al., 2024). To address this limitation, bispecific and tri-specific CAR-T cells targeting multiple B-cell antigens such as CD19, CD20, and CD22 are being explored to reduce the risk of immune escape and enhance therapeutic efficacy (De Oliveira Canedo et al., 2025; Schneider et al., 2021; Dai et al., 2020). However, systematic evaluation of multi-targeted CAR T cells has been limited by the lack of well-characterized cellular models of single (sKO), double (dKO), and triple (tKO) antigen knockout of CD19, CD20, and CD22.

Several groups have adopted alternative strategies such as generating CD19, CD20, and CD22 overexpressing lines in antigen-null background cell lines like K562 and A431 to evaluate CAR specificity and function (Schneider et al., 2021; Schneider et al., 2017). However, these models may not accurately recapitulate the biology of B-cell malignancies, as K562 (a chronic myeloid leukemia cell line) and A431 (an epithelial carcinoma cell line) possess fundamentally different transcriptomic, phenotypic, and signaling profiles as compared to B-cell-derived malignancies. Moreover, the overexpression of B-cell antigens in these non-B-cell lines may fail to represent the surface expression patterns seen in malignant B cells, as these cells will continue to express their respective lineage-specific markers. In addition, lentiviral-mediated overexpression involves random integration of the transgene, which can introduce variability and off-target effects, further complicating the interpretation of functional outcomes (Gurumoorthy et al., 2022). This disparity limits their utility in modeling antigen loss and CAR-T resistance in the relevant hematologic context, underscoring the need for B–cell–relevant knockout systems for more physiologically meaningful preclinical studies.

Several B-cell-derived lines, such as Raji (CD19^+^, CD20^+^, CD22^+^), Daudi (CD19^+^, CD20^+^, CD22^+^), Nalm-6 (CD19^+^, CD22^+^), and Reh (CD19^+^, CD22^+^), have been used in various studies to investigate CAR-T activity and antigen escape. (Wang et al., 2021; Tumaini et al., 2013). While these lines offer valuable insights for specific scenarios of antigen loss, they do not collectively provide all the permutations of antigen loss required for rigorous evaluation of bi- and tri-specific CAR constructs. Among them, Raji stands out as an ideal model system due to its endogenous high expression of all three key B-cell antigens CD19, CD20, and CD22, enabling the systematic generation of defined sKO, dKO, and tKO variants within the same genetic and cellular background. This consistency offers a controlled and reproducible platform to dissect the mechanisms of CAR-T resistance and antigen escape under clinically relevant conditions.

The use of antigen knockout models derived from single-cell clones ensured complete and stable antigen ablation within a consistent genetic background. This clonal precision improves the reliability and reproducibility of functional assays. Additionally, most studies are limited to sKOs, whereas in this study, we have developed a complete panel of single (sKO), double (dKO), and triple (tKO) knockout Raji lines, all from the same parental background. This allows systematic and side-by-side testing of mono-, bi-, and tri-specific CAR-T therapies under consistent conditions, providing a robust platform to evaluate antigen escape and optimize multi-antigen targeting strategies.

Clinical reports have documented dual antigen loss, wherein patients relapsing after CD19 CAR-T therapy and subsequently treated with CD20 or CD22 CAR-T cells experienced further relapse associated with loss of the second target antigen (Duell et al., 2024; Shalabi et al., 2018; Yang et al., 2024). Our double-knockout lines directly model this dual antigen loss scenario and therefore provide a relevant platform for improved screening and selection of CAR targets. Importantly, our aim was not limited to modelling only reported antigen loss scenarios but to generate cell line models encompassing all possible combinations of CD19, CD20, and CD22 loss. This enables more robust evaluation of CAR-T cells designed to target multiple antigens simultaneously, thereby addressing clinically observed patterns of relapse due to antigen escape. The tKO model, which simultaneously lacks CD19, CD20, and CD22, acts as a more physiologically relevant negative control system for studying complete antigen escape in a uniform genetic background.

We utilized a CRISPR/Cas9-mediated genome editing approach to knock out antigen expression of CD19, CD20, and CD22 in Raji cells, simulating the antigen-negative relapse observed in patients undergoing CAR-T cell therapy. sgRNAs for each gene were designed in Exon 1 or 2 to ensure complete loss of protein expression by preventing the production of truncated or partially functional antigen variants. The use of paired sgRNAs to generate deletions in both alleles further ensured an irreversible loss of antigen expression, reducing the likelihood of alternative splicing or re-expression. These genomic deletions were confirmed by amplicon-based targeted next-generation sequencing. Knockout validation was further performed at both transcript and protein levels. qRT-PCR analysis demonstrated negligible transcript levels for the CD19 and CD20 genes in their respective KO lines. Notably, CD22 transcripts, although markedly reduced compared to Raji Luc-WT, were still detectable in CD22 KO cells. This residual expression mirrors observations in relapsed patients with CD22 antigen escape, where CD22 mRNA remains detectable despite a lack of functional surface expression (Davila and Brentjens, 2016; Westin et al., 2023). Protein-level analyses using immunofluorescence and flow cytometry confirmed the complete absence of the respective antigens on the cell surface, establishing the KO lines as effective models that closely mimic antigen loss.

To enable both *in vitro* and *in vivo* assessment of CAR-T cell cytotoxicity, we established a clonal luciferase-expressing Raji cell line to ensure that all cells carried a uniform copy number of the luciferase gene. This reporter system was generated prior to knockout line development in the parental Raji cells, providing a consistent background for eliminating variability in luminescence-based measurements. Incorporating luciferase facilitates a streamlined assessment of cytotoxicity across both *in vitro* and *in vivo* environments. The luciferase assay offered a quantitative and high-throughput approach to assess CAR-T cell-mediated killing with greater precision than other traditional endpoint assays. Moreover, bioluminescence imaging (BLI) would allow for real-time, non-invasive tumor tracking following luciferin administration, enabling longitudinal monitoring of tumor burden in mouse models. This standardized system would ensure reliable and reproducible cytotoxicity assessments across experimental conditions.

Subsequently, we demonstrate that the engineered Raji KO cell lines serve as an effective model for studying antigen escape in CAR-T cell therapy. By using mono-specific second-generation CAR constructs targeting CD19, CD20, and CD22, we confirmed that CAR-T cells exhibit dose-dependent cytotoxicity against wild-type targets, highlighting robust and specific antitumor activity. Importantly, the lack of killing observed in CD19-null, CD20-null, and CD22-null Raji cells with these mono-specific CAR T cells validates that the KO cell lines reliably simulate immune evasion, representing a critical escape mechanism often seen in clinical settings. These findings align with clinical observations where antigen-negative clones persist and expand following CAR-T cell infusion, ultimately leading to therapeutic failure (Lin et al., 2024).

Unlike patient-derived relapse samples, which are limited and typically require prolonged waiting periods for the initiation and follow-up studies of CAR-T trials, our antigen knockout cellular models provide an immediate and reproducible alternative platform to study antigen loss dynamics under controlled conditions. These models can be further refined by integrating *in vivo* xenograft studies to evaluate the clinical relevance of antigen-negative tumor persistence. Mixing these defined knockout lines in varying ratios enables the generation of *in vivo* tumor models that better reflect clinical reality, where heterogeneous tumor clones often express different combinations of antigens (Schneider et al., 2021).

In conclusion, we established a robust cellular platform featuring multiple combinations of antigen knockouts, effectively mimicking clinically relevant patterns of antigen loss in B-cell malignancies. This will aid in the rigorous functional validation of all therapeutic permutations targeting CD19, CD20, and CD22, which are considered the most clinically significant antigens in B-cell malignancies. These models enable systematic, side-by-side evaluation of mono-, bi-, and tri-specific CAR-T constructs within a consistent genetic background. This platform addresses a critical gap in preclinical immunotherapy research and supports the rational development of next-generation, multi-targeted CAR-T strategies.

## Data Availability

The datasets presented in this study can be found in online repositories. The names of the repository/repositories and accession number(s) can be found in the article/Supplementary Material.
