# Supplementary material for: CRISPR-mediated generation of a tumor-associated antigen-deficient Raji platform to investigate antigen loss in CAR-T cell therapy
Source: Front Genome Ed. 2025 Sep 29;7:1649993. doi: 10.3389/fgeed.2025.1649993 (PMC12515939; doi:10.3389/fgeed.2025.1649993)

Supplementary Material

# Supplementary Figures


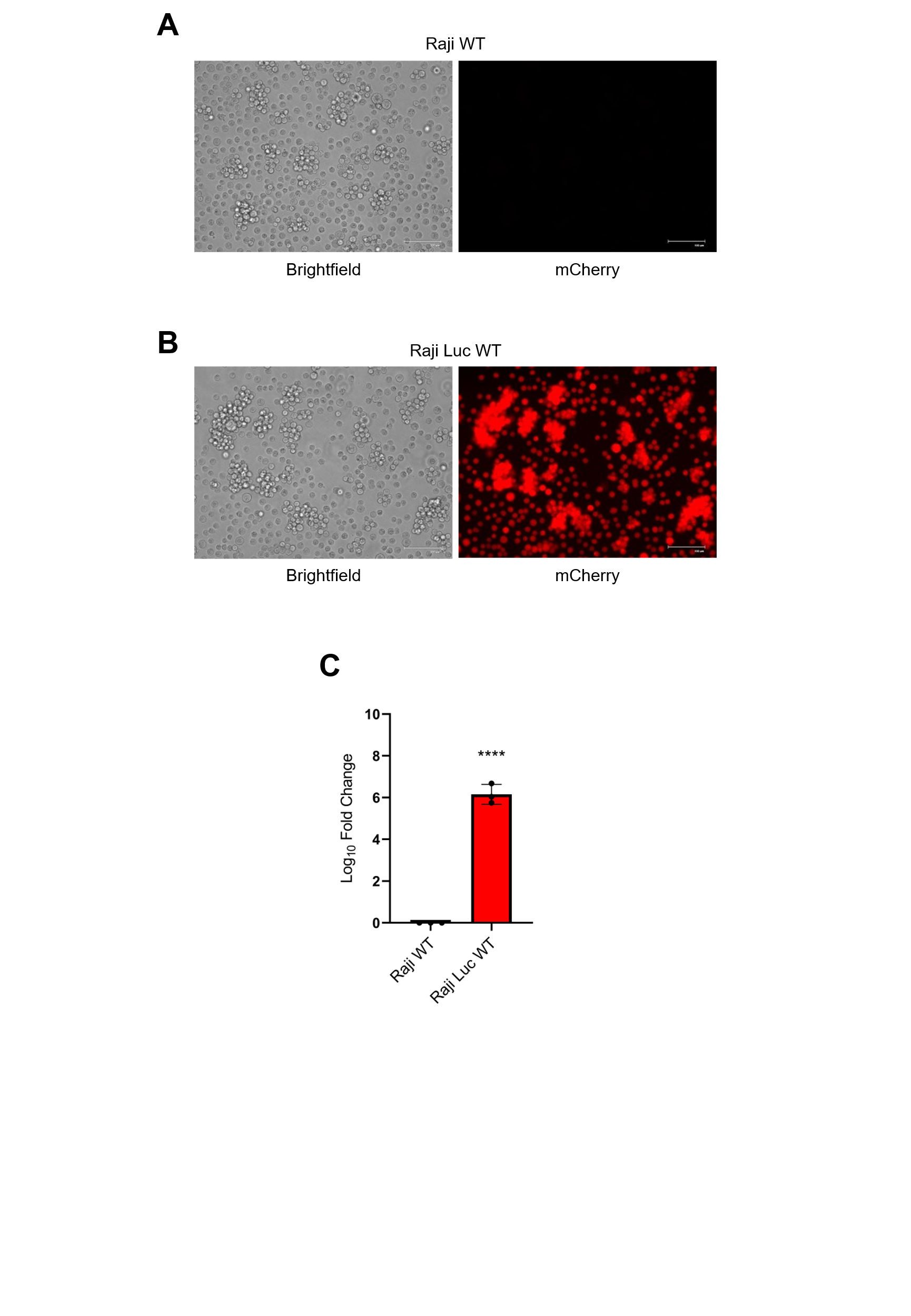


**Supplementary Figure S1. Raji-Luc WT exhibit stable and robust transgene expression**

(A, B) Fluorescence microscopy imaging of Raji WT (A) and Raji-Luc WT (B) show mCherry expression only in the transduced Raji-Luc WT cells. Brightfield images are shown alongside for reference. (C) Raji-Luc WT cells exhibit a significant increased expression (~ 10^6^ fold ) of luciferase transcript levels as revealed by qRT-PCR analysis compared to Raji WT, where the expression was undetectable. GAPDH was used as housekeeping control. Data are presented as mean ± SD for three independent biological replicates. Statistical significance was determined by using two tailed student’s t-test. ****p < 0.0001.


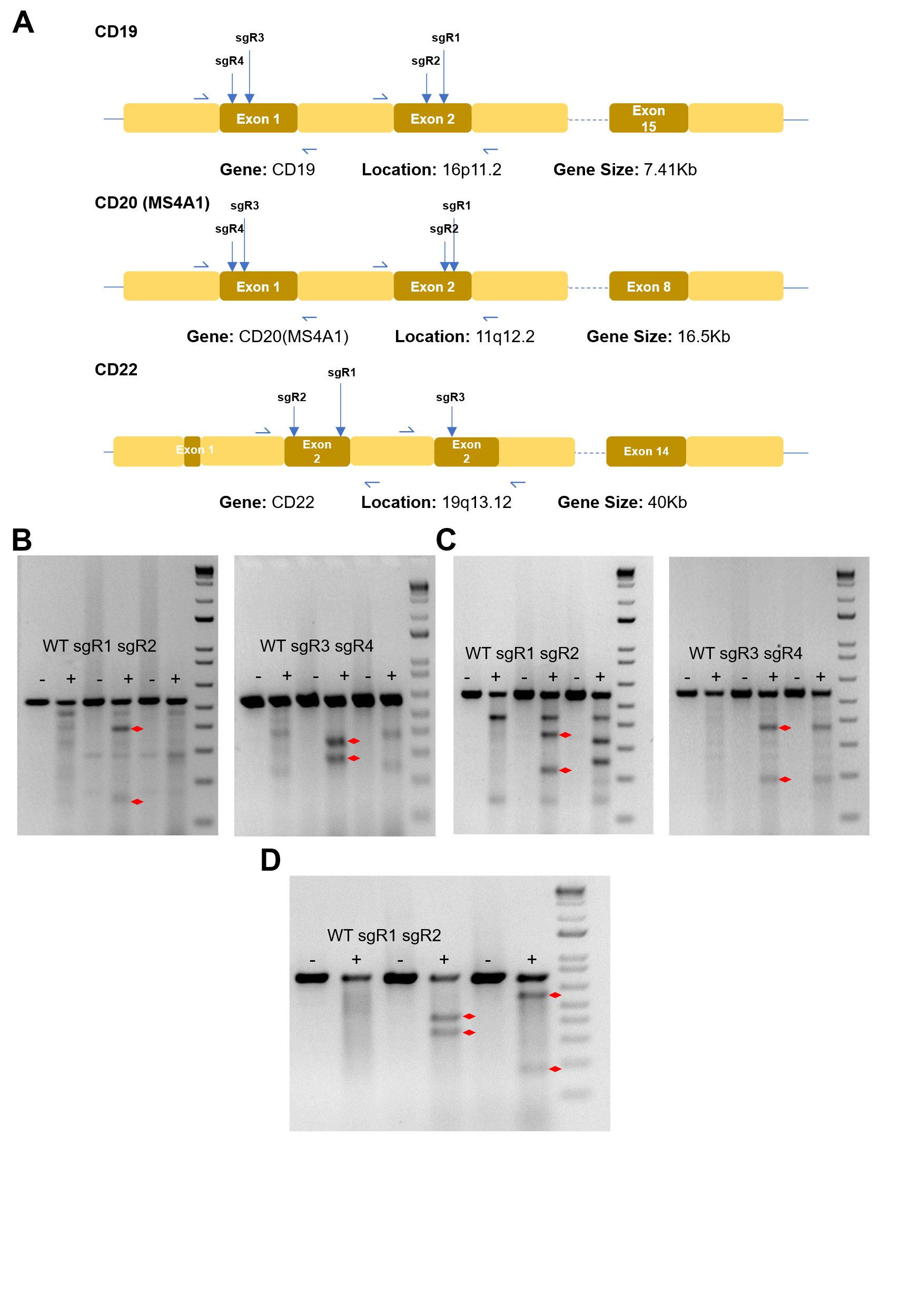


**Supplementary Figure S2. sgRNA Design and Validation for Targeting CD19, CD20, and CD22**

(A) Schematic representation of CD19, CD20 (MS4A1), and CD22 gene loci with exon organization and positions of designed sgRNAs(Blue arrow) and genotyping primers(blue half-arrow) used for CRISPR-Cas9 mediated targeting. (B–D) T7 endonuclease I assay for individual sgRNAs targeting CD19 (B), CD20 (C), and CD22 (D). Red marks indicate cleaved DNA fragments, confirming Cas9-induced indels and validating sgRNA cutting efficiency. WT: wild-type unedited control.


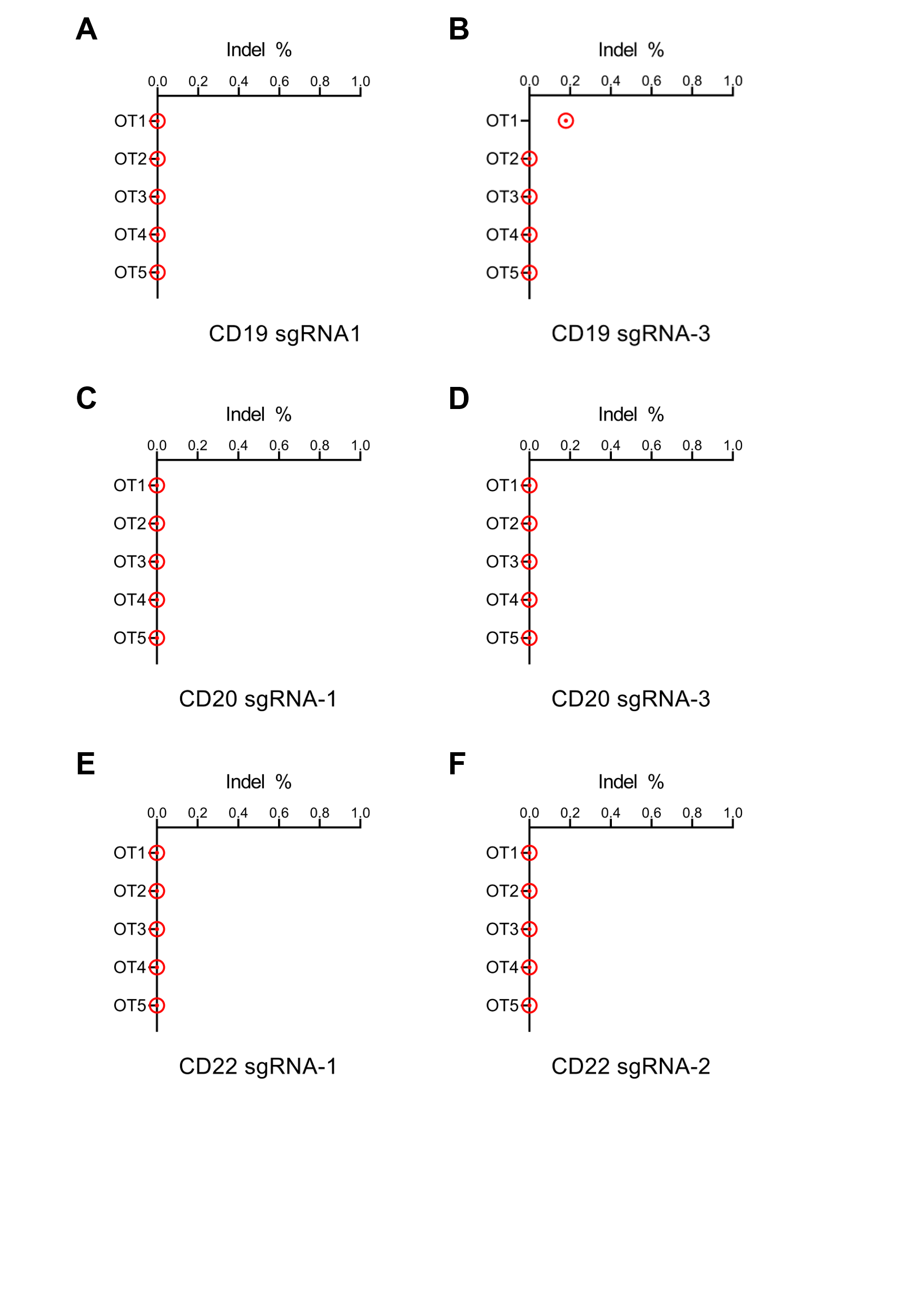


**Supplementary Figure S3: Off-target assessment of the sgRNAs**

Top five COSMID-predicted off-target sites for each of the two sgRNAs targeting CD19 (A, B), CD20 (C, D), and CD22 (E, F) were assessed by NGS-based amplicon sequencing. All sgRNAs showed negligible off-target activity, with only CD19 sgRNA-3 displaying a minimal indel rate (0.18%) at a single site (OT1).


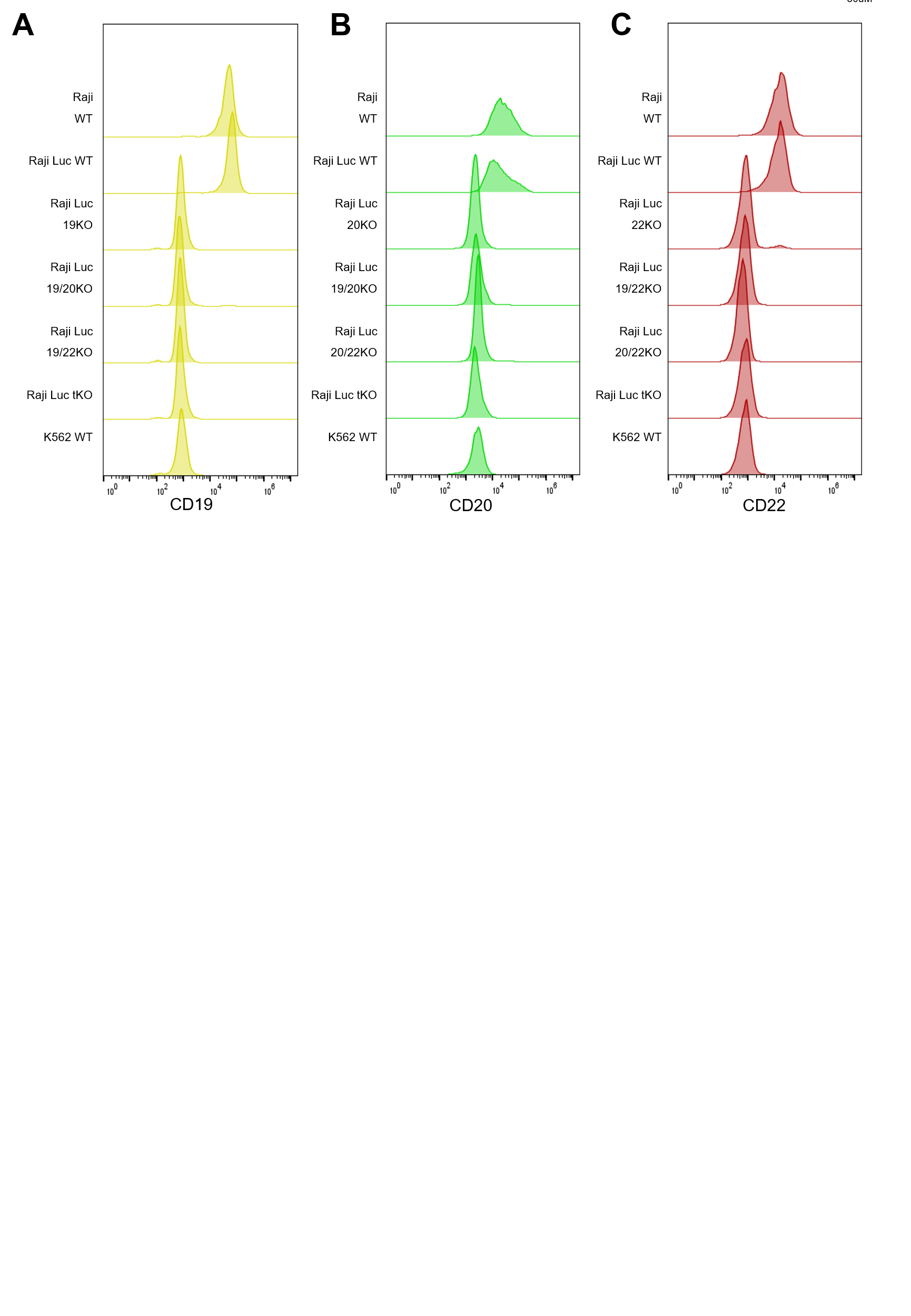


**Supplementary Figure S4: Representative Flow Cytometry peaks for Raji knockouts**

(A–C) Flow cytometry data of surface CD19 (A), CD20 (B), and CD22 (C) expression in the Raji-derived KO lines and K562 control confirms complete loss of target antigen surface expression in respective knockout lines. Histogram shown is the representative data from three independent experiments.


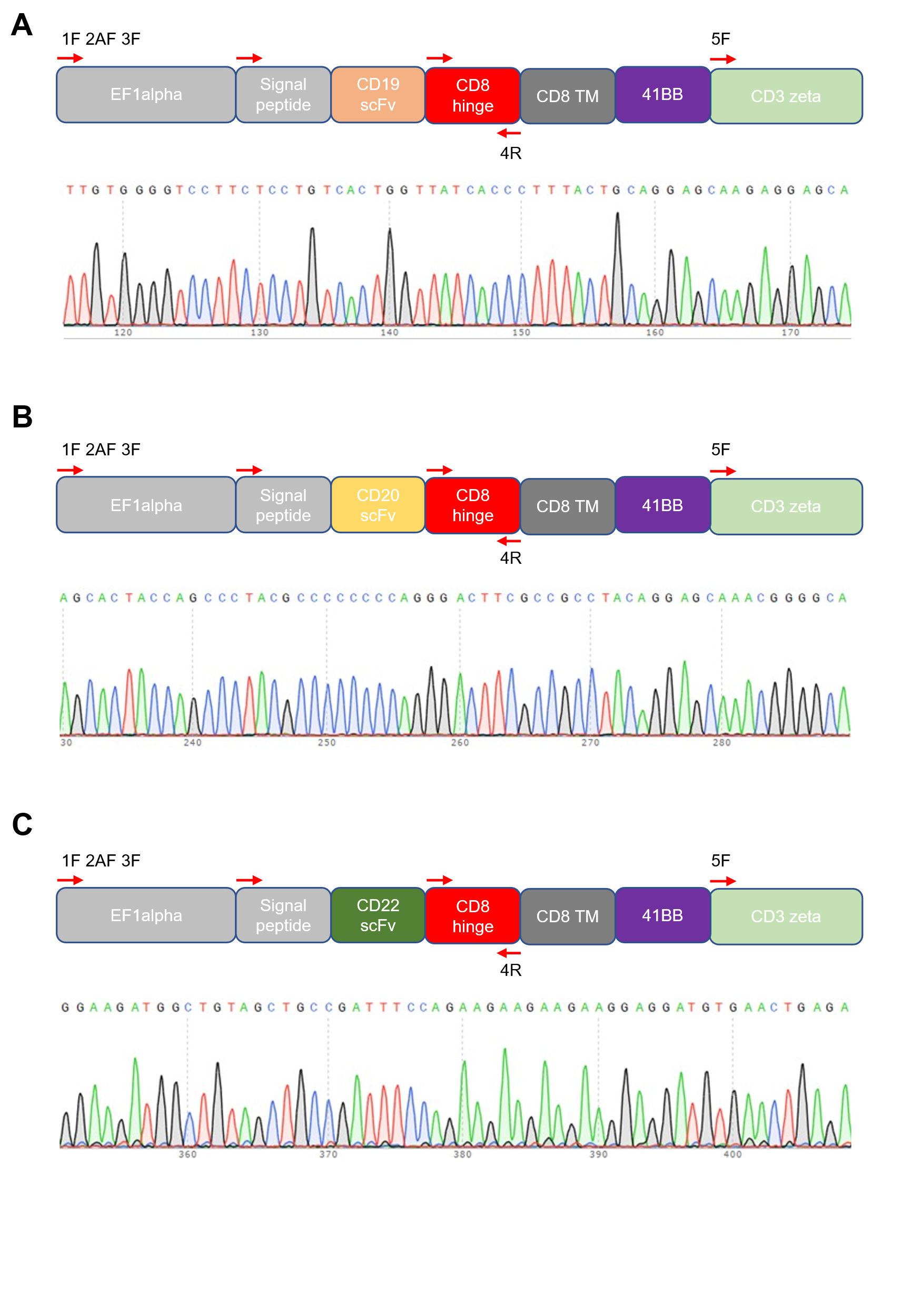


**Supplementary Figure S5: Sanger Sequencing Validation of CAR Constructs**

Schematic representation and corresponding Sanger sequencing chromatograms confirming correct assembly of CAR constructs targeting CD19 (A), CD20 (B), or CD22 (C). Red arrows indicate primer binding sites used for Sanger sequencing


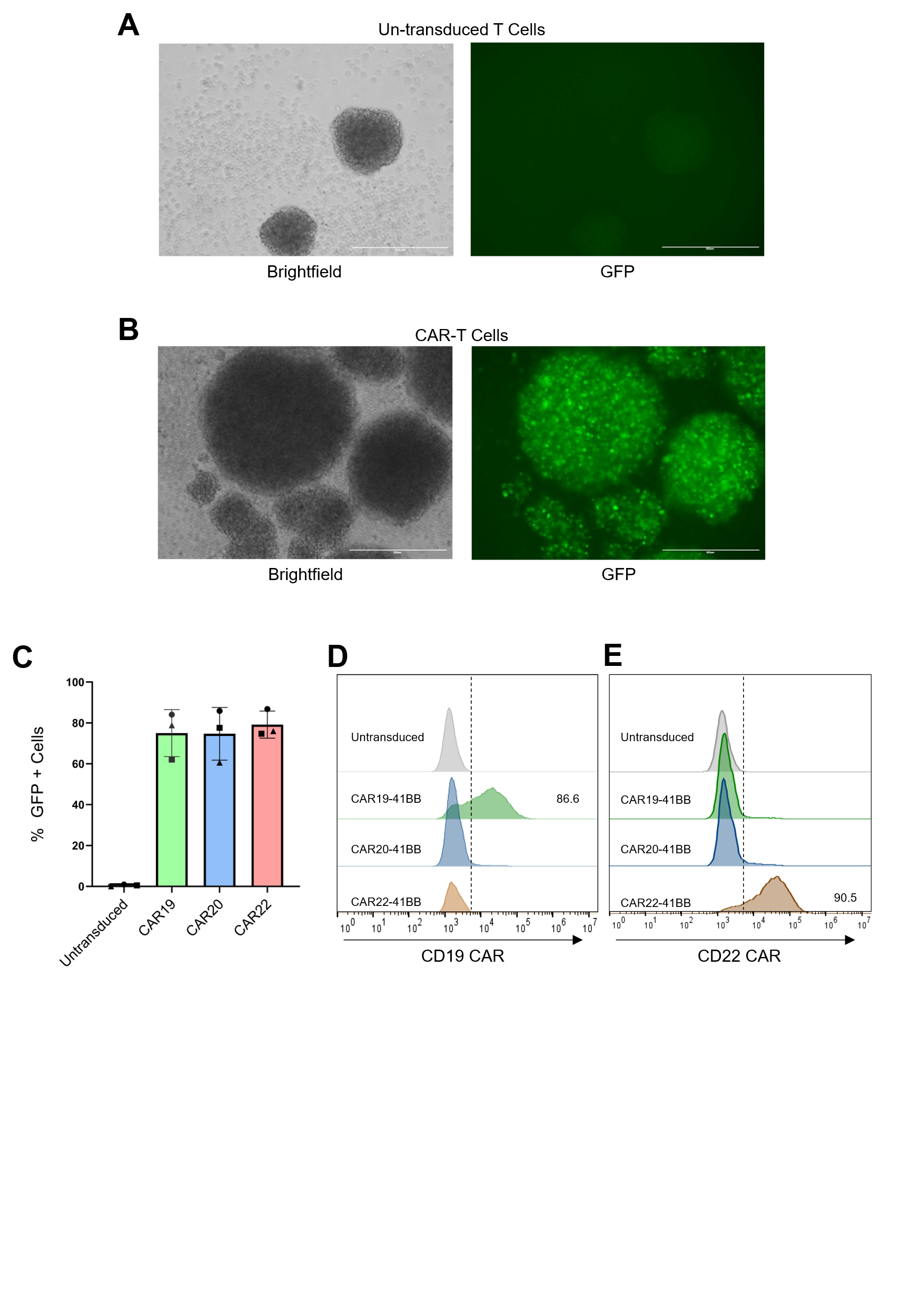


**Supplementary Figure S6: Validation of T Cell Transduction by assessing GFP and CAR Surface Expression**

(A, B) Representative brightfield and GFP fluorescence images of untransduced (A) and CAR-transduced T cells. (C) Percentage of GFP+ cells measured by flow cytometry from three independent transductions (Data plotted as Mean ± SD). (D, E) Flow cytometry data of scFv surface expression of CAR19 (D) and CAR22 (E) in CAR-T cells and untransduced control confirms antigen specificity of CAR scFvs. Histogram shown is the representative data from three independent experiments.

# Supplementary Tables


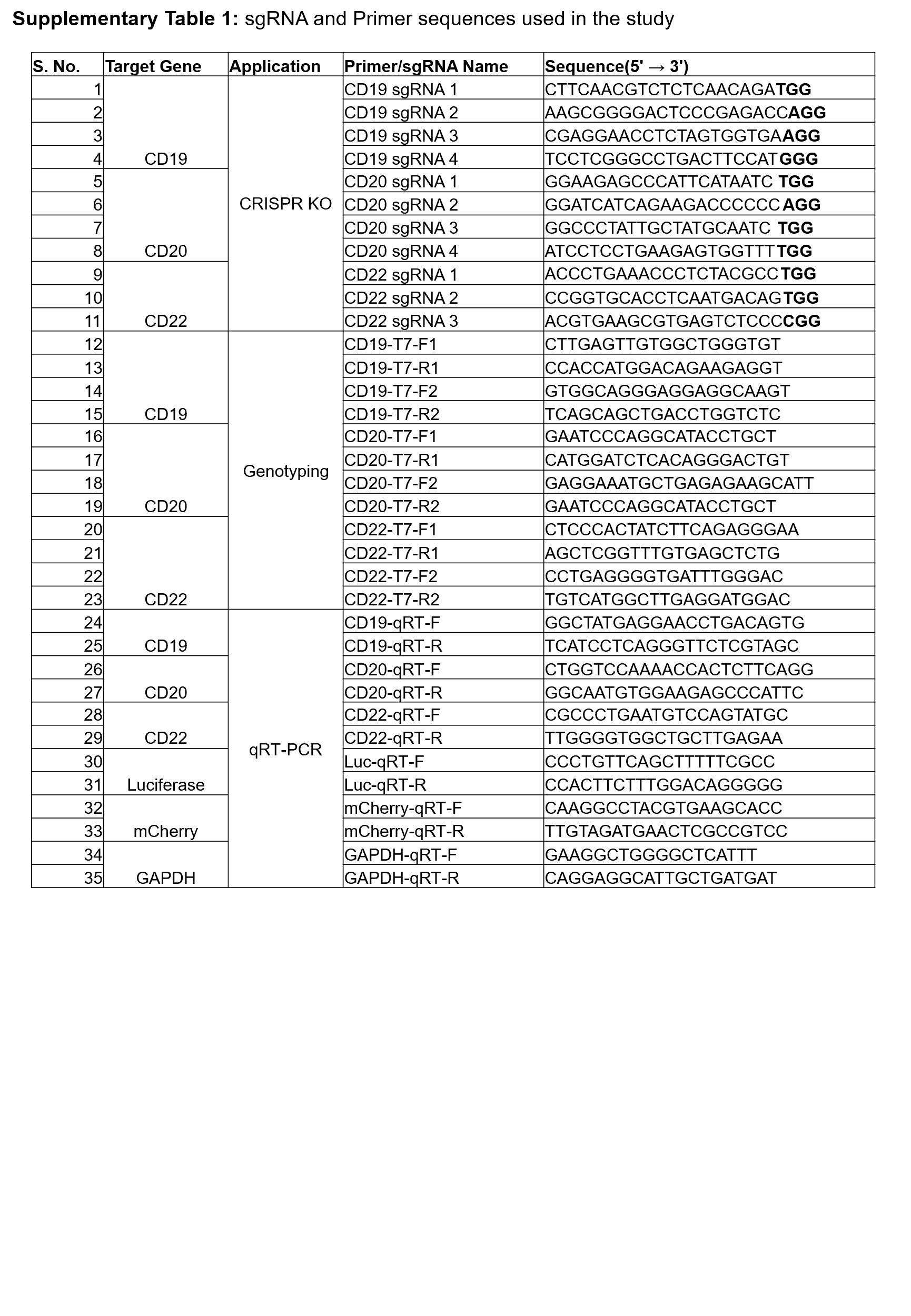
**Supplementary Table 1:** sgRNA and Primer sequences used in the study


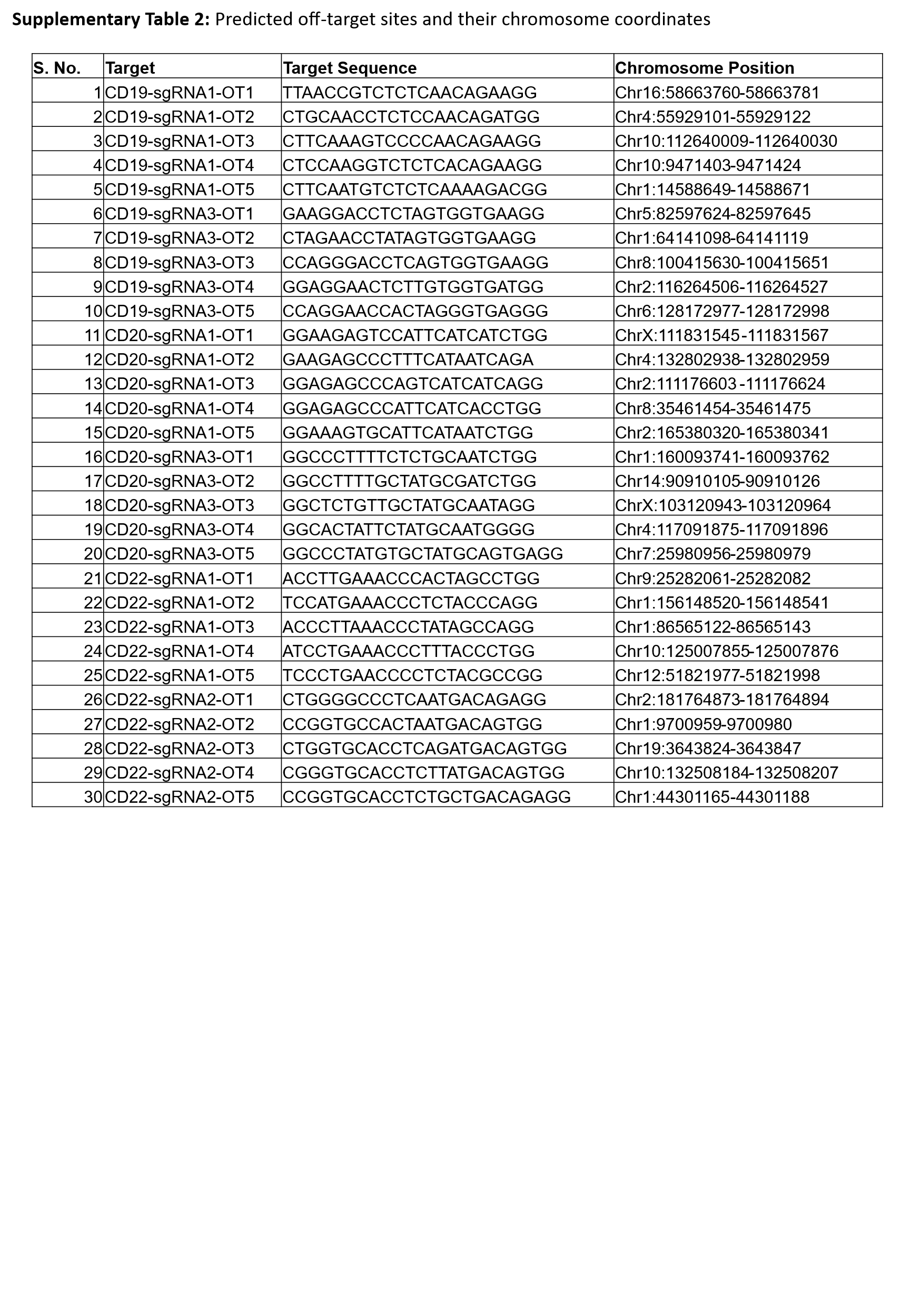
**Supplementary Table 2:** Predicted off-target sites and their chromosome coordinates

**Supplementary Table 3:** Antibodies and cytokines used in the study


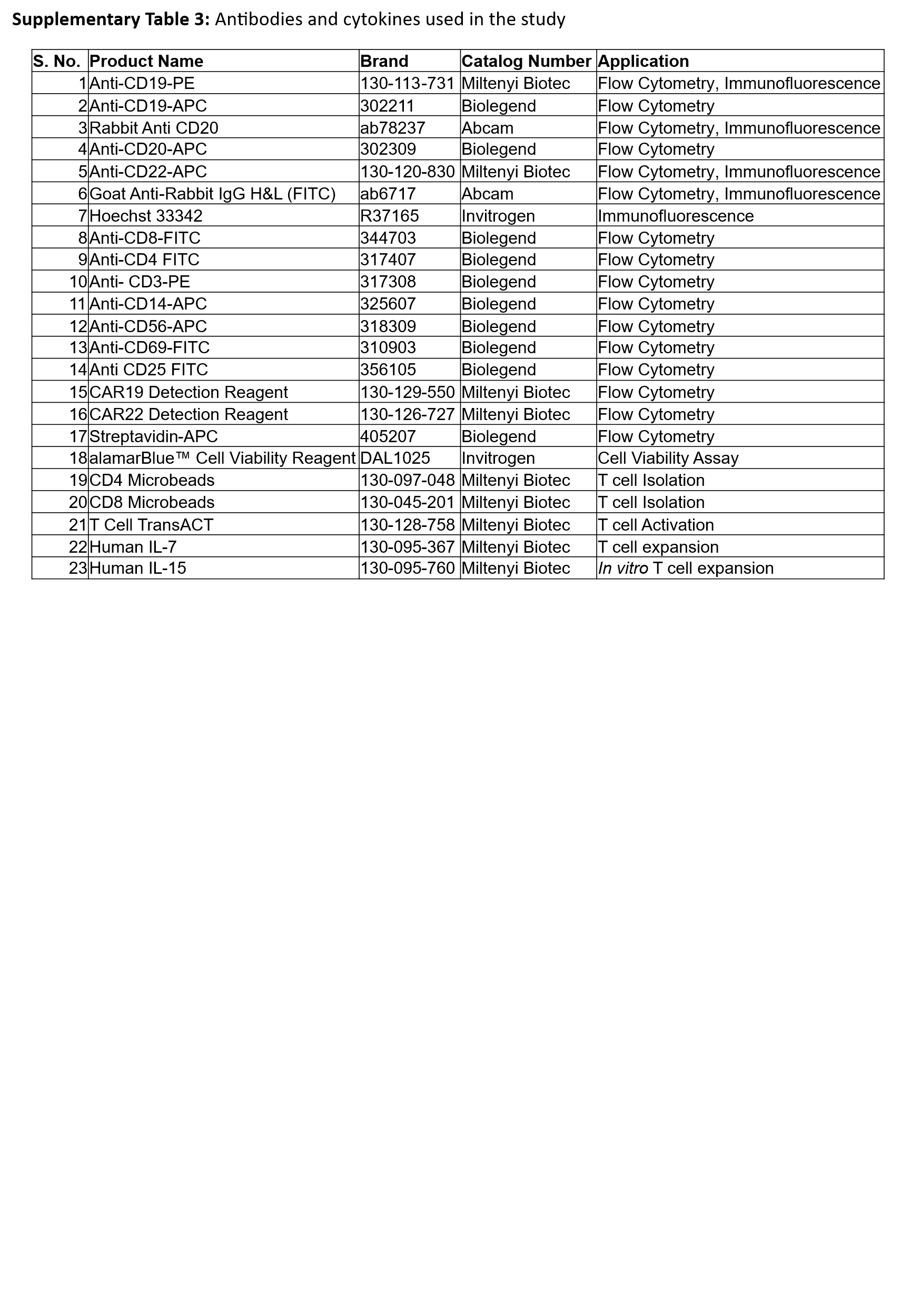

Supplement: Supplementary file 1 [file Supplementaryfile1.docx]
